# Maxillary Incisive Canal Characteristics: A Radiographic Study Using Cone Beam Computerized Tomography

**DOI:** 10.1155/2019/6151253

**Published:** 2019-03-27

**Authors:** Penala Soumya, Pradeep Koppolu, Krishnajaneya Reddy Pathakota, Vani Chappidi

**Affiliations:** ^1^Department of Dentistry, Mahavir Institute of Medical Sciences, Vikarabad, Telangana, India; ^2^Department of Preventive Dental Sciences, College of Dentistry, Dar Al Uloom University, Riyadh, Saudi Arabia; ^3^Department of Peroiodontics, Sri Sai College of Dental Surgery, Vikarabad, India; ^4^Department of Oral Medicine and Radiology, Sri Sai College of Dental Surgery, Vikarabad, Telangana, India

## Abstract

**Background:**

The incisive canal located at the midline, posterior to the central incisor, is an important anatomic structure of this area to be considered while planning for immediate implant placement in maxillary central incisor region. The purpose of the present study is to assess incisive canal characteristics using CBCT sections.

**Materials and Methods:**

CBCT scans of 79 systemically healthy patients, with intact maxillary incisors, were evaluated by two calibrated and independent examiners. Assessments included (1) mesiodistal diameter, (2) labiopalatal diameter, (3) length of the incisive canal, (4) shape of incisive canal, and (5) width of the bone anterior to the incisive foramen.

**Results:**

The mean width of the foramen labiopalatally and mesiodistally was 3.12 ± 0.94 mm and 3.23 ± 0.98 mm, respectively. Mean canal length was 18.63 ± 2.35 mm and males have significantly longer incisive canal than females. The mean width of bone anterior to the incisive canal was 6.32 ± 1.43 mm. As age of the subjects increased, incisive foramen diameter and incisive canal length were found to be increased. Cylindrical shaped incisive canals were seen in most of the individuals followed by funnel shaped and hour-glass shaped canals, and banana-like canal is least prevalent type.

**Conclusion:**

The findings from the present study suggest that the diameter and length of incisive canal vary among different individuals and presence of very thin bone anterior to the canal would suggest that a pretreatment CBCT scan is a valuable tool to evaluate anatomic variations, morphology, and dimensions of incisive foramen before immediate implant placement in maxillary central incisor region.

## 1. Introduction

Immediate implant placement is gaining much of clinical importance in recent years and requires a thorough knowledge of important anatomic structures in concerned area. Implant placement in maxillary central incisor region is often challenging due to anatomical variations in dimensions of incisive canal and foramen. Incisive canal has two openings: incisive foramen and nasopalatine foramen [1]. The nasopalatine nerves and vessels traverse through this canal. Placement of implant into incisive canal results in nervous tissue injury, sensory dysfunction, and nonosseointegration of implant.

Several morphological alterations in the incisive canal shape have been described; Mardinger et al. [2] classified incisive canal shape as cylindrical, funnel like, banana like, and hour-glass like. Size, shape, position, and number of foramina vary in different individuals. Presence of wider foramina and thin alveolar bone anterior to the canal may make an implant placement almost impossible in this area.

Careful evaluation and planning are necessary before an immediate implant placement in anterior maxilla. Clinically sound and sophisticated radiograph techniques such as dental CTs can assist in diagnosing deficiencies. Preoperative CBCT of maxillary incisor region helps in diagnosing any anatomical difficulties before proceeding with an implant placement. CBCT is noninvasive, has high resolution, low dose of radiation, and financial advantage, and allows full 3D characterization of alveolar bone.

The aim of this study was to determine maxillary incisive canal characteristics in relation to the maxillary central incisors using CBCT images.

## 2. Materials and Methods

Hundred systemically healthy dentulous and/or partially edentulous patients (53 males and 47 females) aged between 17 and 72 years (with a mean age of 42.7 years) scheduled for implant insertion in different dental clinics in Hyderabad were included in the study. Written informed consent was obtained from all the patients and the Ethical Committee of Sri Sai College of Dental Surgery approved this study. Subjects with missing maxillary incisors, with evident nasopalatine pathology, and images with poor quality are excluded.

All CBCT scans were obtained with 1 mm slice thickness and the tomographic scanner CS 9000C 3D CBCT scanner with exposure settings of 120 kV, 15 mA, and 12-inch field of view was used to obtain CBCT scan. A software program, Care stream 3D Imaging software, was used to reconstruct the images and perform the measurements.

### 2.1. Interexaminer Calibration

Two observers were calibrated using 10 randomly selected scans. An assessment of the reproducibility of measurement between observers measuring the same quantity to one-tenth of a millimeter was calculated at a correlation of 0.95 for the 10 scans. Each of the two observers measured 79 scans independently at the exact same slice and magnification.

### 2.2. Parameters Evaluated

**Figure 1:**
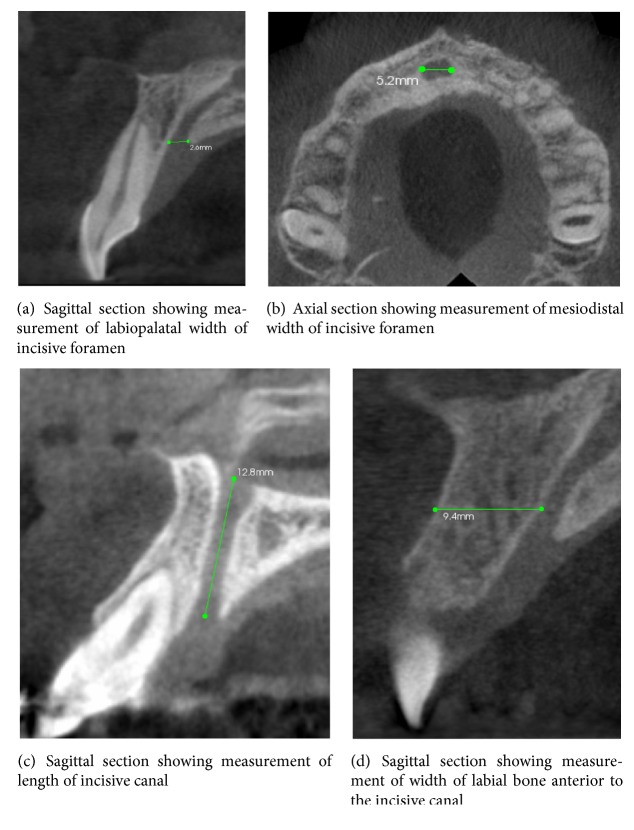


**Figure 2:**
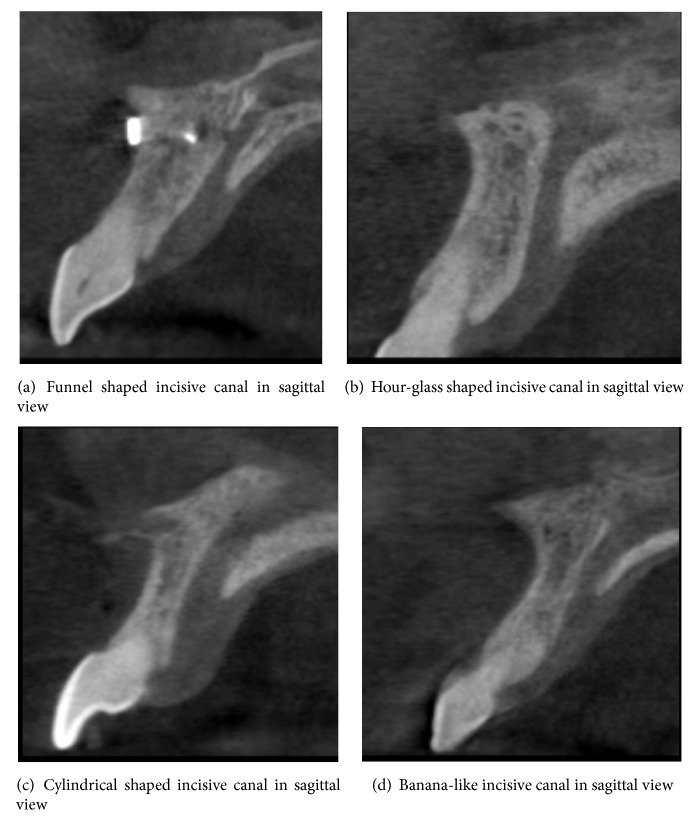


The following characteristics of incisive were evaluated:

1. Width of the nasopalatine canal labiopalatally and mesiodistally (Figures 1(a) and 1(b))
2. Length of the canal (Figure 1(c))
3. Width of the bone anterior to the canal (Figure 1(d))
4. Shape of the canal (Figures 2(a)–2(d))

Comparison of characteristics between males and females and correlation with age is done.

## 3. Statistical Analysis

Mean and standard deviations (SD) were calculated for all the variables. All statistical analysis was performed using SPSS version 18.0. Independent sample t test was used to compare the incisive canal characteristics between male and female subjects. Analysis of variance (ANOVA) was used to evaluate the influence of age on the incisive canal characteristics. A p value <0.05 was considered to be statistically significant and a p value <0.001 is considered to be highly statistically significant.

## 4. Results

Initially CT scans of 100 patients were assessed, of which 21 were excluded. A total of 79 scans were evaluated for the determined parameters. Mean length of canal was 18.63 ± 2.35 mm. Mean diameter of the nasopalatine canal mesiodistally was 3.23 ± 0.98 mm, and labiopalatally it was 3.12 ± 0.94 mm. Mean width of the bone anterior to the foramen was found to be 6.32 ± 1.43 mm (Table 1).

When the influence of age on the canal length was assessed, it was observed to be increasing with age. Younger individuals are found to have significantly smaller length of incisive canal when compared to older individuals and this was found to be statistically significant (Table 2). When males and females were compared, i.e., when the effect of gender was evaluated, there was no significant difference between male and female subjects (Table 2).

When the effect of age on the diameter of incisive canal was evaluated, there was a statistically significant (p<0.05) increase in diameter of incisive foramen both labiopalatally and mesiodistally (Table 2) and when effect of gender was evaluated, there was no significant difference between males and females (Table 3).

Bone width anterior to the canal was in the range of 4 to 10.4 mm with a mean width of 6.32 ± 1.43 mm; when different age groups were compared, with increasing age there seems to be a decrease in mean width of bone anterior to the incisive canal, although it was not statistically significant (p=0.2) (Table 2).

Male subjects had a mean bone width of 6.50 ± 1.52 mm and female subjects had a width of 6.10 ± 1.32 mm anterior to the incisive canal. Females had a thin bone plate anterior to the incisive canal when compared to males, which was not statistically significant (Table 1).

Of the 79 scans evaluated most prevalent shape of the canal was found to be cylindrical followed by funnel shaped canal, hour-glass like canal, and banana-like canals (Figures 2(a)–2(d)).

## 5. Discussion

Close proximity of incisive canal to maxillary central incisor region and a thin anterior labial bone may sometimes hamper the immediate implant placement or one may end up in encroachment of the canal leading to sensory dysfunction and nonosseointegration. Careful planning and evaluation using CBCT help in diagnosing such anatomical deficiencies.

Several studies mentioned the anatomic features of this area. In the present study incisive canal characteristics were measured using CBCT sections. To the best of the authors' knowledge this was the first study evaluating the incisive canal characteristics using CBCT sections in Indian subjects.

Mean canal length was found to be 18.63 ± 2.35 mm, which is longer when compared to other studies. Tozum et al. [3] in their multicentered trial found a mean canal length of 10.86 ± 2.67. Liang et al. [4] examined 120 spiral CTs and found a length of 9.9 ± 2.6 mm. Song et al. [1] have demonstrated that mean length of canal in dentate maxillae is 12 mm (8.4 mm to 15.6 mm). Mraiwa et al. [5] reported a mean length of 8.1 ± 3.4 mm. The longer incisive canal length seen in the present study compared to other studies can be attributed to variations in the anatomical characteristics in Indian population.

Mean canal diameter measured 3.23 ± 0.98 mm and 3.12 ± 0.94 mm, mesiodistally and labiopalatally, respectively. In a similar study Tozum et al. [3] measured mean foramen diameter at two different points, i.e., superior and inferior orifice, and it was found to be 2.76 ± 1.40 mm and 2.93 ± 1.01 mm. In the present study foramen width was measured in two different CBCT sections (axial and sagittal sections) and the measurements were comparatively larger than Tozum et al. [3] study; this can be attributed to variations in the anatomical characteristics in Indian population.

Immediate implant placement is advantageous as it decreases the time elapse between tooth loss and restoration and reduces the number of surgical procedures. Initial stability of an implant placed in maxillary incisor area is primarily dependent on the width of the bone anterior to the canal and length of the bone apical to the roots. Although augmentation procedures can be done to modify the width of bone anterior to the canal, it is a determinant of dimensions of implant in that area. In the present study width of bone anterior to the canal was found to be 6.32 ± 1.43 mm. This value is in accordance with Tozum et al. [3] study, where they have found a mean width of 7.38 ± 1.42 mm.

In the present study no significant correlation was detected between gender and incisive canal characteristics. Many studies have reported gender influence on incisive canal characteristics (Mraiwa et al. [5], Mardinger et al. [2], Liang et al. [4], Song et al. [2], Bornstein et al. [6], Kovisto et al. [7], and Tozum et al. [3]). Mraiwala et al. [5] found no significance relation between gender and incisive canal characteristics, which is in accordance with the present study. Bornstein et al. [6] found higher canal length in males when compared to females. Liang et al. [4] reported greater canal length and width in males when compared to female subjects. Guncu et al. [8] examined CTs of 417 male and 516 female patients and concluded that there are statistically significant gender related differences in anatomical features of incisive canal like canal length, diameter, and bone thickness anterior to the incisive canal.

Age of the patient had a significant influence on the length of the incisive canal and diameter of incisive foramen; with increasing age there was an increase in incisive canal length and diameter of incisive foramen. Similarly, Bornstein et al. [6] reported that age of the subjects had a significant influence on the length of the incisive canal. However, Tozum et al. [3] and Mriawa et al. [5] could not find any relation between age and incisive canal characteristics.

Incisive canal morphology was classified in various ways. Mardinger et al. [2] classified the shape of the incisive canals based on how they look in cross sectional view of CBCT scans into cylindrical, funnel shaped, hour-glass shaped, and banana-like canals. In the present study the most predominant shape of the canal was cylindrical shaped followed by funnel shaped, hour-glass shaped, and banana-like. These results are in accordance with Guncu et al. study [8] in which the predominant canal shape was cylindrical; they further analyzed percentage of different shapes of incisive canal in males and females. Similarly Kajan et al. [9] reported predominance of cylindrical shaped incisive canal. In contrast to these studies Etoz et al. [10] examined 500 CBCT scans of dentate patients and found predominance of hour-glass shaped incisive canals.

In edentulous patients with severe atrophic maxilla, invasive procedures like removal of neurovascular bundle from the incisive canal and subsequent grafting of the incisive canal, followed by placement of dental implants, have been presented [11, 12]. A recent clinical study proposed removal of neurovascular bundle from the incisive canal and subsequent placement of implant in seven patients, with only few patients experiencing minor complications [13].

This signifies the importance of assessing incisive foramen characteristics before planning for implant placement; a clinician requires a thorough knowledge of incisive canal anatomical variations. CBCT is an advanced diagnostic aid which helps in diagnosing anatomical difficulties before planning for implant placement in maxillary incisor area.

## 6. Conclusion

Within the limitations of the study, the present study suggests that the diameter and length of incisive canal vary among different individuals and presence of very thin bone anterior to the canal would suggest that a pretreatment CBCT scan is a valuable tool to evaluate anatomic variations, morphology, and dimensions of incisive foramen before immediate implant placement in maxillary central incisor region. Findings from this study suggest that age has a significant influence over incisive canal characteristics.

## Figures and Tables

**Table 1:** Demographic variables and descriptive statistics of 79 individuals.

Descriptive statistics	Mean ± SD
Age	42.36 ± 16.44 years
Canal length	18.63 ± 2.35 mm
Mesiodistal width	3.23 ± 0.98 mm
Labiopalatal width	3.12 ± 0.94 mm
Width of the bone anterior to the canal	6.32 ± 1.43 mm

**Table 2:** Comparison of incisive canal characteristics according to age using ANOVA.

Parameter	<30 years (n=23)	31-50 (n=23)	>50 (n=33)	p value
Canal length	14.7 ± 1.41	16.93 ± 1.25	19.13 ± 1.61	<0.0001^*∗*^
Mesiodistal width	3 ± 0.75	2.92 ± 0.89	3.71 ± 1.07	0.003^*∗*^
Labiopalatal width	2.81 ± 0.98	2.86 ± 0.87	3.6 ± 0.83	0.001^*∗*^
Width of the bone anterior to the canal	6.73 ± 1.67	6.3 ± 1.33	6.02 ± 1.34	0.2

Values are presented as mean ± standard deviation.

^*∗*^Statistically significant difference between the age groups.

**Table 3:** Comparison of incisive canal characteristics between male and female subjects.

Parameter	Male (n=43)	Female (n=36)	p value
Canal length	19.07 ± 1.09	18.38 ± 2.87	0.72
Mesiodistal width	3.25 ± 1.05	3.21 ± 0.92	0.42
Labiopalatal width	3.23 ± 0.89	2.99 ± 1.0	0.13
Width of the bone anterior to the canal	6.50 ± 1.52	6.10 ± 1.32	0.11

Values are presented as mean ± standard deviation.

## Data Availability

The data used to support the findings of this study are available from the corresponding author upon request.
